# Three new amide derivatives from the fungus *Alternaria brassicicola*

**DOI:** 10.1007/s13659-023-00391-2

**Published:** 2023-09-11

**Authors:** Fengli Li, Saisai Gu, Sitian Zhang, Shuyuan Mo, Jieru Guo, Zhengxi Hu, Yonghui Zhang

**Affiliations:** 1 Hubei Key Laboratory of Natural Medicinal Chemistry and Resource Evaluation, School of Pharmacy, Tongji Medical College, Huazhong University of Science and Technology, Wuhan, 430030 China; 2 Department of Pharmacy, Union Hospital, Tongji Medical college, Huazhong University of Science and Technology, Wuhan, 430022 China; 3 Department of Pharmacy, Tongji Hospital, Tongji Medical college, Huazhong University of Science and Technology, Wuhan, 430033 China

**Keywords:** *Alternaria brassicicola*, Secondary metabolites, Amide derivatives, Structural elucidation

## Abstract

Three new amide derivatives (alteralkaloids A–C, **1**–**3**) and three known alkaloids (**4**–**6**) were afforded after phytochemical investigation of fungus *Alternaria brassicicola*. The structures of these compounds were confirmed by NMR spectroscopic and HRESIMS data. Furthermore, the absolute configuration of **1** was determined using the single-crystal X-ray diffraction analysis. Compounds **1**–**3** belong to a class of amide derivatives that have not been found in nature before, sharing the same characteristic signals of the butyl moiety and amide group. These isolated compounds mentioned above were tested for the cytotoxic activity.

**Graphical Abstract:**

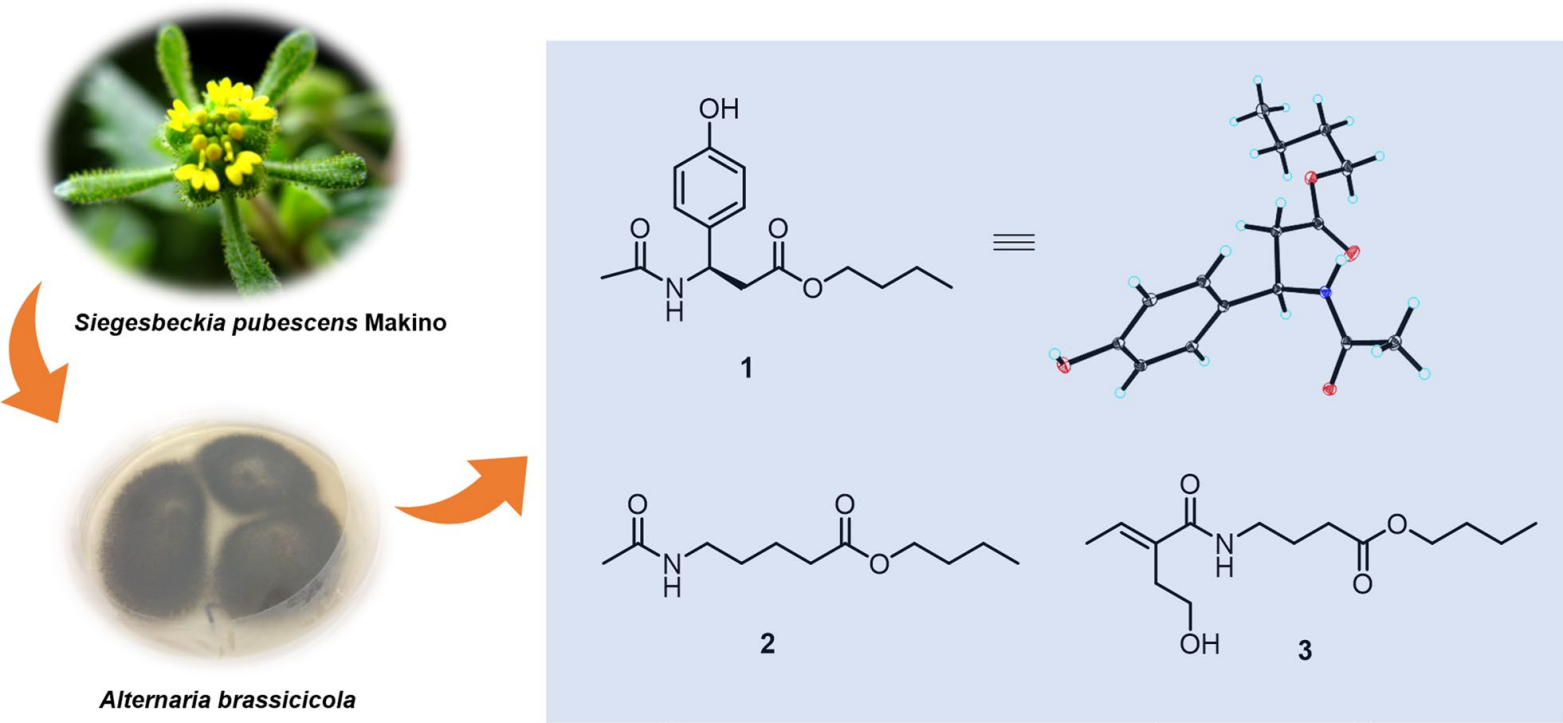

**Supplementary Information:**

The online version contains supplementary material available at 10.1007/s13659-023-00391-2.

## Introduction

*Alternaria* fungi are one of the important biological resources with great potentials, from which a rich source of novel structures with a variety of bioactivities were discovered successively [1–3]. They mainly included nitrogen-containing metabolites [4], diterpenoids [5], meroterpenoids [6], and polyketides [7, 8]. And these metabolites exhibited a broad range of biological activities, such as anti-inflammatory [9], phytotoxic [10], cytotoxic [11], acetylcholinesterase inhibitory [12], and antimicrobial activities [13], which attracted, and still attract increasing attentions from the chemists and pharmacologists.

**Fig. 1:**
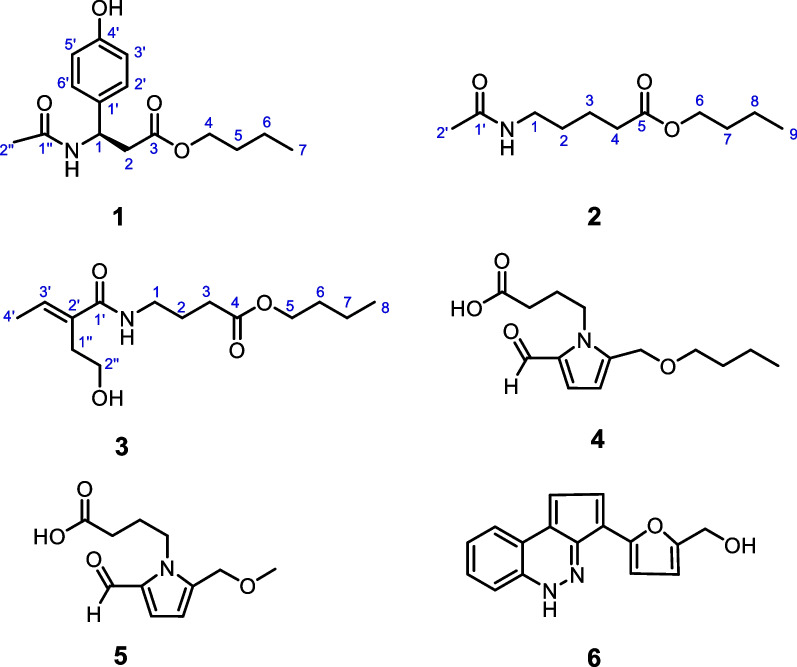
Chemical structures of compounds **1**–**6**

During the chemical investigation of structurally interesting and biologically active constituents from fungus *A. brassicicola*, our group have identified a variety of novel structures. Notably, brassicicene N represented the first fusicoccane-derived diterpenoid with a tetracyclic skeleton bearing an oxabicyclo[4.3.1]nonane unit [14]; alterbrassicene A possessed an unprecedented 5/9/4-fused carbocyclic skeleton, exhibiting potent IKK*β* inhibitory effect [9]; alterbrassicicene A, featuring a newly transformed monocyclic carbon skeleton, showed strong agonistic action upon PPAR-*γ* [15]; alterbrassinoids A–D represented the first class of fusicoccane-derived diterpenoid dimers [16]. Based on our previous work, we amplified and fermented this potential fungus again. As a result, three new compounds (**1**–**3**) along with three known alkaloids (**4**–**6**) were afforded in the present phytochemical study on the fungus *A. brassicicola*. Herein, this paper aims to report the isolation, structural elucidation, and the cytotoxic activity of these isolated compounds (Fig. 1).

## Results and discussion

**Fig. 2:**
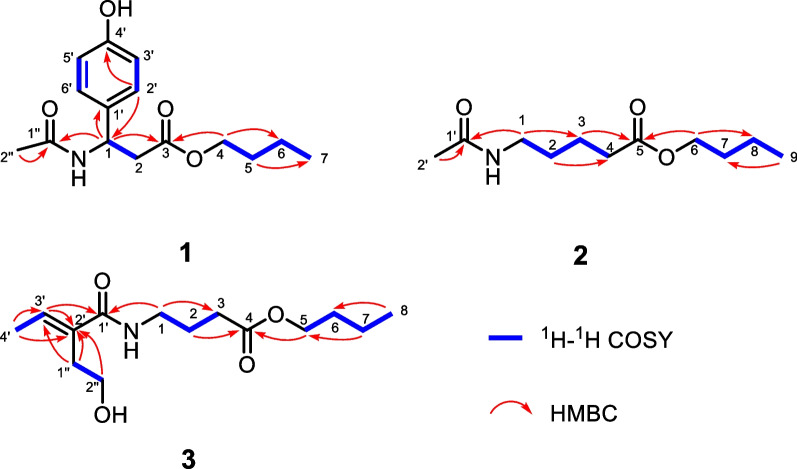
Key ^1^H–^1^H COSY and HMBC correlations of compounds **1**–**3**

**Fig. 3:**
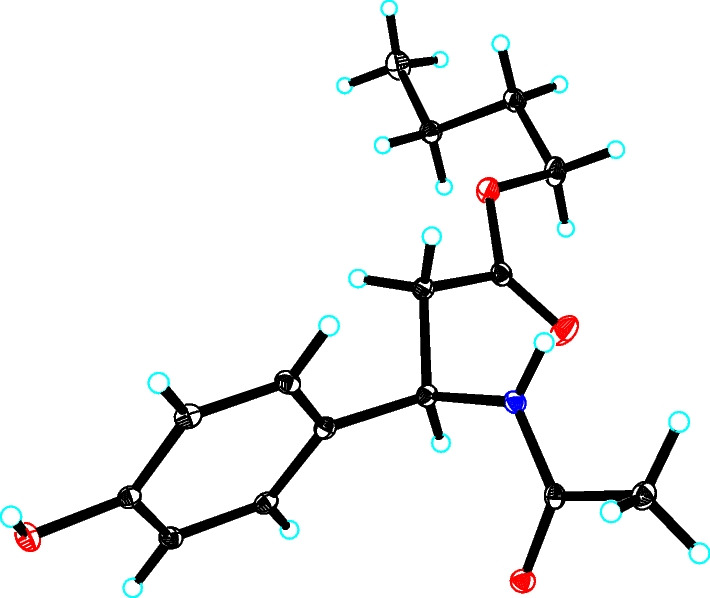
X-Ray crystallographic structure of **1**

Compound **1** was isolated as a colorless needle crystal, and its molecular formula was confirmed as C_15_H_21_NO_4_ based on the HRESIMS data at *m/z* 302.1378 (Additional file 1). According to the characteristic signals of the 1D NMR data (Table 1) showing H-2′/H-6′ (*δ*_H_ 7.15, d *J* = 8.5 Hz) and H-3′/H-5′ (*δ*_H_ 6.74, d, *J* = 8.5 Hz); and C-1′ (*δ*_C_ 133.2), C-2′/C-6′ (*δ*_C_ 128.9), C-3′/C-5′ (*δ*_C_ 116.3), and C-4′ (*δ*_C_ 158.0), the *para*-substituted benzene ring can be deduced. The spin coupling system (Fig. 2) of H_3_-7 (*δ*_H_ 0.9)/H_2_-6 (*δ*_H_ 1.31)/H_2_-5 (*δ*_H_ 1.53)/H_2_-4 (*δ*_H_ 4.02) suggested the existence of a butyl fragment. The spin coupling system (Fig. 2) of H-1 (*δ*_H_ 5.25)/H_2_-2 (*δ*_H_ 2.79), along with the HMBC correlations (Fig. 2) of H_2_-4 and H-1 with C-3 (*δ*_C_ 172.4) and of H-2′ with C-1 (*δ*_C_ 51.2), suggested that the butyl moiety was connected to the 3-(4-hydroxyphenyl)propanoic acid group. In addition, the HMBC correlations from H_3_-2″ (*δ*_H_ 1.92) and H-1 to C-1″ (*δ*_C_ 172.2) were observed, which confirmed an acetamido motif attached to the C-1. Thus, the planar structure of **1** was corroborated (Fig. 1). The absolute configuration of **1** was confirmed as 1*R* using the single-crystal X-ray diffraction analysis [Flack parameter = 0.01(5), CCDC 2049878] (Fig. 3). Therefore, the structure of **1** was determined and named as alteralkaloid A.

**Table 1:** ^1^H NMR and ^13^C NMR data (*δ* in ppm, *J* in Hz) for **1**–**3**

No.	**1** (in methanol-*d*_4_)		**2** (in methanol-*d*_4_)		**3** (in methanol-*d*_4_)	
	*δ*_C_^a^	*δ*_H_^b^	*δ*_C_^a^	*δ*_H_^b^	*δ*_C_^a^	*δ*_H_^b^
1	51.2 CH	5.25 t (7.6)	40.0 CH_2_	3.17 t (6.6)	39.3 CH_2_	3.23 t (6.9)
2	41.9 CH_2_	2.79 m	29.8 CH_2_	1.52 m	25.9 CH_2_	1.80 m
3	172.4 C		23.4 CH_2_	1.69 m, 1.55 m	32.4 CH_2_	2.36 m
4	65.5 CH_2_	4.02 t (6.6)	34.6 CH_2_	2.34 t (7.3)	175.0 C	
5	31.8 CH_2_	1.53 m	175.3 C		65.4 CH_2_	4.08 t (6.6)
6	20.1 CH_2_	1.31 m	65.3 CH_2_	4.07 t (6.6)	31.8 CH_2_	1.62 m
7	14.0 CH_3_	1.90 t (7.4)	31.8 CH_2_	1.61 m	20.2 CH_2_	1.40 m
8			20.2 CH_2_	1.40 m	14.0 CH_3_	0.95 t (7.4)
9			14.0 CH_3_	0.95 t (7.4)		
1′	133.2 C		173.2 C		169.7 C	
2′	128.9 CH	7.15 d (8.5)	22.5 CH_3_	1.92 s	151.6 C	
3′	116.3 CH	6.74 d (8.5)			120.9 CH	5.71 q (1.4)
4′	158.0 C				18.5 CH_3_	2.12 d (1.4)
5′	116.3 CH	6.74 d (8.5)				
6′	128.9 CH	7.15 d (8.5)				
1″	172.2 C				44.5 CH_2_	2.32 m
2″	22.6 CH_3_	1.92 s			60.8 CH_2_	3.70 t (6.6)

“m” mean overlapped or multiplet with other signals

^a^Recorded at 100 MHz

^b^Recorded at 400 MHz

Compound **2**, obtained as a colorless oil, showed an [M + Na]^+^ peak at *m/z* 238.1473 in the HRESIMS analysis, matching a molecular formula of C_11_H_21_NO_3_, which was indicative of two degrees of unsaturation. Based on its 1D NMR data (Table 1), a total of 11 carbons were observed, from which two methyl groups (*δ*_C_ 14.0 and 22.5), seven methylene groups (*δ*_C_ 20.2, 23.4, 29.8, 31.8, 34.6, 40.0, and 65.3), and two carbonyls (*δ*_C_ 173.2 and 175.3) were included. The characteristic signals [spin coupling system: H_2_-6 (*δ*_H_ 4.07)/H_2_-7 (*δ*_H_ 1.61)/H_2_-8 (*δ*_H_ 1.40)/H_3_-9 (*δ*_H_ 0.95)] of the butyl moiety were also observed in the ^1^H–^1^H COSY spectrum of **2** (Fig. 2). In addition, the spin coupling system of H_2_-1 (*δ*_H_ 3.17)/H_2_-2 (*δ*_H_ 1.52)/H_2_-3 (*δ*_H_ 1.69, 1.55)/H_2_-4 (*δ*_H_ 2.34) along with the HMBC correlations (Fig. 2) from H_2_-3 and H_2_-6 to C-5 (*δ*_C_ 175.3) and from H_3_-2′ (*δ*_H_ 1.92) and H_2_-1 to C-1′ (*δ*_C_ 173.2) were clearly observed, which defined the structure of **2** as butyl 5-acetamidopentanoate and this compound was named as alteralkaloid B (Additional file 1).

Compound **3** was obtained as a colorless oil. Its HRESIMS data at *m/z* 294.1689 indicated that the molecular formula was C_14_H_25_NO_4_, suggesting three degrees of unsaturation. The 1D NMR (Table 1) and HSQC data showed 14 carbon signals, which was similar to that of **2**, including two methyl groups (*δ*_C_ 14.0 and 18.5), eight methylene groups (*δ*_C_ 20.2, 25.9, 31.8, 32.4, 39.3, 44.5, 60.8, and 65.4), two carbonyls (*δ*_C_ 175.0 and 169.7), and two olefinic carbons (*δ*_C_ 120.9 and 151.6). The structure contained one double bond and two carbonyl groups, suggesting that **3** was a chain compound. The ^1^H–^1^H COSY correlations (Fig. 2) of H_2_-1 (*δ*_H_ 3.23)/H_2_-2 (*δ*_H_ 1.80)/H_2_-3 (*δ*_H_ 2.36) and H_2_-5 (*δ*_H_ 4.08)/H_2_-6 (*δ*_H_ 1.62)/H_2_-7 (*δ*_H_ 1.40)/H_3_-8 (*δ*_H_ 0.95), together with the HMBC correlations (Fig. 2) from H_2_-2 and H_2_-5 to C-4 (*δ*_C_ 175.0), suggested the existence of a butyl butyrate fragment (A unit). In addition, two spin coupling systems of H_2_-1″ (*δ*_H_ 2.32)/H_2_-2″ (*δ*_H_ 3.70) and H_3_-4′ (*δ*_H_ 2.12)/H-3′ (*δ*_H_ 5.71) in the ^1^H–^1^H COSY spectrum of **3** (Fig. 2) and the key HMBC correlations (Fig. 2) of H_3_-4′ and H_2_-1″ with C-3′ (*δ*_C_ 120.9) and C-2′ (*δ*_C_ 151.6), indicated the existence of a 2-(2-hydroxyethyl)but-2-enamide fragment (B unit). The deduction that C-1 of A unit was connected to the nitrogen atom of B unit was supported by the HMBC correlation from H_2_-1 to C-1′ (*δ*_C_ 169.7). To sum up, the structure of **3** was confirmed and named as alteralkaloid C.

Three known compounds were confirmed as lobechine (**4**) [17], 4-[2-formyl-5-(methoxymethyl)-1*H*-pyrrol-1-yl]butanoic acid (**5**) [18], and 2-furanmethanol-(5′→11)-1,3-cyclopentadiene-[5,4-*c*]-1*H*-cinnoline (**6**) [19] by comparing their NMR data with the literatures.

Compounds **1**–**6** were tested for the cytotoxicity against six cell lines, including five human cancer cell lines (HepG2, Hep3B, HT-29, HeLa, and OCVAR) and the normal liver cell LO2. Unfortunately, no compound showed obvious cytotoxicity against the above cell lines (IC_50_ > 40 µM) (Additional file 1).

### Supplementary Information

**Additional file 1.** Includes 1D and 2D NMR, HRESIMS, UV, and IR of compounds **1**–**3**.
