# Supplementary material for: Three new amide derivatives from the fungus Alternaria brassicicola
Source: Nat Prod Bioprospect. 2023 Sep 11;13(1):28. doi: 10.1007/s13659-023-00391-2 (PMC10495297; doi:10.1007/s13659-023-00391-2)
Supplement: Supplementary file 1 — Additional file 1. Includes 1D and 2D NMR, HRESIMS, UV, and IR of compounds 1–3. [file 13659_2023_391_MOESM1_ESM.docx]

Additional file 1

**Three** **new amide derivatives from the fungus *Alternaria brassicicola***

Fengli Li^1†^, Saisai Gu^2†^, Sitian Zhang^1^, Shuyuan Mo^1^, Jieru Guo^3*^, Zhengxi Hu^1*^ and Yonghui Zhang^1*^

^1^Hubei Key Laboratory of Natural Medicinal Chemistry and Resource Evaluation, School of Pharmacy, Tongji Medical College, Huazhong University of Science and Technology, Wuhan 430030, China

^2^Department of Pharmacy, Union Hospital, Tongji Medical college, Huazhong University of Science and Technology, Wuhan 430022, China

^3^Department of Pharmacy, Tongji Hospital, Tongji Medical college, Huazhong University of Science and Technology, Wuhan 430033, China

^[[1]](#footnote-1)^

**Contents**

[**1. General experimental procedures** 3](#_Toc99717872)

[**2. Fungal material** 3](#_Toc99717873)

[**3. Extraction and isolation** 3](#_Toc99717874)

[**4. X-ray crystal structure analysis** 5](#_Toc99717875)

[**5. Cytotoxicity assay** 6](#_Toc99717876)

[**Figure S1.** ^1^H NMR spectrum of compound **1** (Recorded in methanol-*d*_4_) 7](#_Toc99717877)

[**Figure S2** ^13^C NMR and DEPT spectrum of compound **1** (Recorded in methanol-*d*_4_) 8](#_Toc99717878)

[**Figure S3.** HSQC spectrum of compound **1** (Recorded in methanol-*d*_4_) 9](#_Toc99717879)

[**Figure S4.** HMBC spectrum of compound **1** (Recorded in methanol-*d*_4_) 10](#_Toc99717880)

[**Figure S5** ^1^H–^1^H COSY spectrum of compound **1** (Recorded in methanol-*d*_4_) 11](#_Toc99717881)

[**Figure S6.** NOESY spectrum of compound **1** (Recorded in methanol-*d*_4_) 12](#_Toc99717882)

[**Figure S7.** HRESIMS spectrum of compound **1** 13](#_Toc99717883)

[**Figure S8.** UV spectrum of compound **1** 14](#_Toc99717884)

[**Figure S9**. IR spectrum of compound **1** 15](#_Toc99717885)

[**Figure S10.** ^1^H NMR spectrum of compound **2** (Recorded in methanol-*d*_4_) 16](#_Toc99717886)

[**Figure S11.** ^13^C NMR and DEPT spectrum of compound **2** (Recorded in methanol-*d*_4_) 17](#_Toc99717887)

[**Figure S12.** HSQC spectrum of compound **2** (Recorded in methanol-*d*_4_) 18](#_Toc99717888)

[**Figure S13.** HMBC spectrum of compound **2** (Recorded in methanol-*d*_4_) 19](#_Toc99717889)

[**Figure S14**. ^1^H–^1^H COSY spectrum of compound **2** (Recorded in methanol-*d*_4_) 20](#_Toc99717890)

[**Figure S15.** HRESIMS spectrum of compound **2** 21](#_Toc99717891)

[**Figure S16.** UV spectrum of compound **2** 22](#_Toc99717892)

[**Figure S17**. IR spectrum of compound **2** 23](#_Toc99717893)

[**Figure S18.** ^1^H NMR spectrum of compound **3** (Recorded in methanol-*d*_4_) 24](#_Toc99717894)

[**Figure S19.** ^13^C NMR and DEPT spectrum of compound **3** (Recorded in methanol-*d*_4_) 25](#_Toc99717895)

[**Figure S20.** HSQC spectrum of compound **3** (Recorded in methanol-*d*_4_) 26](#_Toc99717896)

[**Figure S21.** HMBC spectrum of compound **3** (Recorded in methanol-*d*_4_) 27](#_Toc99717897)

[**Figure S22**. ^1^H–^1^H COSY spectrum of compound **3** (Recorded in methanol-*d*_4_) 28](#_Toc99717898)

[**Figure S23.** HRESIMS spectrum of compound **3** 29](#_Toc99717899)

[**Figure S24.** UV spectrum of compound **3** 30](#_Toc99717900)

[**Figure S25**. IR spectrum of compound **3** 31](#_Toc99717901)

**Experimental Section**

# **1. General experimental procedures**

Optical rotations were tested from a Perkin-Elmer 341 spectropolarimeter (Waltham, MA, USA) in MeOH. UV spectra were collected from a Varian Cary 50 instrument (Santa Clara, CA, USA) in MeOH. IR spectra were recorded on a Vertex 70 FT-IR spectrophotometer (Billerica, MA, USA) with KBr pellets. CD data were recorded on a JASCO-810 spectrometer (Oklahoma City, OK, USA) in MeOH. HRESIMS data were collected from a Bruker MicrOTOF II spectrometer using electrospray ionization (Bruker, Germany). 1D & 2D NMR spectra were tested on a Bruker AM-400 spectrometer. The chemical shifts (δ) were expressed in ppm with reference to the solvent signals for methanol-d_4_ (δ_H_ 3.31 and δ_C_ 49.0). Semi-preparative HPLC was carried out on Agilent HPLC system with a reversed-phase (RP) C_18_ column (5 μm, 10 × 250 mm, Welch Ultimate XB-C_18_). Column chromatography (CC) was performed on silica gel (80–120 mesh and 200–300 mesh; Qingdao Marine Chemical Inc., Qingdao, China), and Sephadex LH-20 (Pharmacia Biotech AB, Uppsala, Sweden). Fractions were monitored by TLC and spots were visualized by heating silica gel plates sprayed with 10% H_2_SO_4_ in EtOH.

# **2. Fungal material**

The strain Alternaria brassicicola was isolated from fresh leaves of Siegesbeckia pubescens Makino (Compositae), which was collected from Huoshan County of Anhui Province, China, in September of 2012. This strain was authenticated based on the morphology and the sequence analysis of the ITS region of rDNA (Genbank No. KR779774) and was stored in the culture collection of the College of Pharmacy, Tongji Medical College, Huazhong University of Science and Technology.

# **3. Extraction and isolation**

The fungus *Alternaria brassicicola* was cultured on potato dextrose agar (PDA) plates at 28 °C for five days. Subsequently, the agar was inoculated into 100 × 1000 mL Erlenmeyer flasks of 200 g rice, 200 mL distilled water which were sterilized by autoclave. After 21 days, each flask was added 300 mL of ethanol to stop the growth of fungi and the fermented material was extracted with N-butyl alcohol for three times. The organic solvent was evaporated under vacuum to obtain a crude extract (100 g), which was then fractionated by silica gel column chromatography (CC) eluted with CH_2_Cl_2_–CH_3_OH gradient system (200:1→0:100) to obtain five fractions (Fr. A−E) based on TLC analysis. Fraction B (1 g) was chromatographed on Sephadex LH-20 eluting with MeOH to obtain three fractions (B1−B3). Fraction B1 (250 mg) was subjected to silica gel CC eluted with petroleum ether (PE)−EtOAc (5:1−0:1) progressively and further purified by using semipreparative HPLC to obtain compound **4** (18.0 mg, MeCN−H_2_O, 44:56, v/v, 2.0 mL/min, *t*_R_ = 30 min), **5** (8.8 mg, MeOH−H_2_O, 42:56, v/v, 2.0 mL/min, *t*_R_ = 23 min) and **6** (8.4 mg, MeOH−H_2_O, 66:34, v/v, 2.0 mL/min, *t*_R_ = 32 min). Fraction C (5 g) was chromatographed on an ODS RP-C18 column with gradient elution of MeOH−H_2_O (20%, 40%, 60%, 80%, 100%) to obtain six fractions (C1−C6). Fraction C2 (500 mg) purified by Sephadex LH-20 (MeOH) and RP-HPLC to yield **2** (12.0 mg; MeOH–H_2_O, 56:44, v/v, 2.0 mL/min, *t*_R_ = 30 min) and **3** (10.2 mg; MeOH–H_2_O, 55:45, v/v, 2.0 mL/min, *t*_R_ = 28 min). Fraction C5 (200 mg) was applied to silica gel CC (200–300 mesh) with a gradient elution of PE/EtOAc (5:1→0:1) and further purified by RP-HPLC to yield **1** (6.0 mg; MeCN–H_2_O, 32:68, v/v, 2.0 mL/min, *t*_R_ = 43 min)

Compound **1**: Colorless needle crystals; C_15_H_21_NO_4_; [*α*]25 D: +69 (*c* 1 MeOH); UV (MeOH) *λ*_max_ (log *ε*): 201 (4.52), 216 (3.79), 228 (3.93); ECD (MeOH) *λ*_max_ (Δ*ε*): 206 (+9.26), 213 (+5.53), 225 (+10.56); IR (KBr) *ν*_max_: 3394, 2960, 1733, 1623, 1535, 1517, 1451, 1382, 1234, 1174, 1026, 827; HRESIMS *m/z* [M + Na]^+^ 302.1378 (calcd for C_15_H_21_NO_4_Na^+^, 302.1363). ^1^H NMR (400 MHz, CD_3_OD) data: *δ*_H_ 5.25 (t, *J* = 7.6 Hz, H-1), 2.79 (m, H-2), 4.02 (t, *J* = 6.6 Hz, H-4), 1.53 (m, H-5), 1.31 (m, H-6), 1.90 (t, *J* = 7.4 Hz, H-7), 7.15 (d, *J* = 8.5 Hz, H-2' and H-6'), 6.74 (d, *J* = 8.5 Hz, H-3' and H-5'), 1.92 (s, H-2''); ^13^C NMR (100 MHz, CD_3_OD) data: *δ*_C_ 51.2 (C, C-1), 41.9 (CH_2_, C-2), 172.4 (C, C-3), 65.5 (CH_2_, C-4), 31.8 (CH_2_, C-5), 20.1 (CH_2_, C-6), 14.0 (CH_3_, C-7), 133.2 (C, C-1'), 128.9 (CH, C-2'), 116.3 (CH, C-3'), 158.0 (C, C-4'), 116.3 (CH, C-5'), 128.9 (CH, C-6'), 172.2 (C, C-1''), 22.6 (CH_3_, C-2'') (Table 1).

Compound **2**: Colorless oil; C_11_H_21_NO_3_; UV (MeOH) *λ*_max_ (log *ε*): 205 (3.62); IR (KBr) *ν*_max_: 3387, 3300, 2960, 2936, 1735, 1654, 1555, 1458, 1372, 1171, 1030, 603; HRESIMS *m/z* [M + Na]^+^ 238.1473 (calcd for C_11_H_21_NO_3_Na^+^, 238.1414). ^1^H NMR (400 MHz, CD_3_OD) data: *δ*_H_ 3.17 (t, *J* = 6.6 Hz, H-1), 1.52 (m, H-2), 1.69 (m, H-3a), 1.55 (m, H-3b), 2.34 (t, *J* = 7.3 Hz, H-4), 4.07 (t, *J* = 6.6 Hz, H-6), 1.61 (m, H-7), 1.40 (m, H-8), 0.95 (s, H-9), 1.92 (s, H-2'); ^13^C NMR (100 MHz, CD_3_OD) data: *δ*_C_ 40.0 (CH_2_, C-1), 29.8 (CH_2_, C-2), 23.4 (CH_2_, C-3), 34.6 (CH_2_, C-4), 175.3 (C, C-5), 65.3 (CH_2_, C-6), 31.8 (CH_2_, C-7), 20.2 (CH_2_, C-8), 14.0 (CH_3_, C-9), 173.2 (C, C-1'), 22.5 (CH_3_, C-2') (Table 1).

Compound **3**: Colorless oil; C_14_H_25_NO_4_; UV (MeOH) *λ*_max_ (log *ε*): 221 (4.47); IR (KBr) *ν*_max_: 3382, 2959, 2936, 1734, 1662, 1632, 1545, 1450, 1257, 1176, 1035, 584; HRESIMS *m/z* [M + Na]^+^ 294.1689 (calcd for C_14_H_25_NO_4_Na^+^, 294.1676). ^1^H NMR (400 MHz, CD_3_OD) data: *δ*_H_ 3.23 (t, *J* = 6.9 Hz, H-1), 1.80 (m, H-2), 2.36 (m, H-3), 4.08 (t, *J* = 6.6 Hz, H-5), 1.62 (m, H-6), 1.40 (m, H-7), 0.95 (t, *J* = 7.4 Hz, H-8), 5.71 (q, *J* = 1.4 Hz, H-3'), 2.12 (d, *J* = 1.4 Hz, H-4'), 2.32 (m, H-1''), 3.70 (t, J = 6.6 Hz, H-2''); ^13^C NMR (100 MHz, CD_3_OD) data: *δ*_C_ 39.3 (CH_2_, C-1), 25.9 (CH_2_, C-2), 32.4 (CH_2_, C-3), 175.0 (C, C-4), 65.4 (CH_2_, C-5), 31.8 (CH_2_, C-6), 20.2 (CH_2_, C-7), 14.0 (CH_3_, C-8), 169.7 (C, C-1'), 151.6 (C, C-2'), 120.9 (CH, C-3'), 18.5 (CH_3_, C-4'), 44.5 (CH_2_, C-1''), 60.8 (CH_2_, C-2'') (Table 1).

# **4. X-ray crystal structure analysis**

Crystal of compound **1** were obtained in MeOH. Intensity data were obtained at 296 K on a Bruker APEX DUO diffractometer equipped with an APEX-II CCD using Cu K*α* radiation. Bruker SAINT was used for cell refinement and data reduction. The structures were solved by direct methods with SHELXL-2014/7 [1]. The hydrogen atoms were located in the calculated positions and refined by a riding model. Molecular graphics were computed with PLATON. The crystallographic data (CCDC 2049878 for **1**) have been deposited in the Cambridge Crystallographic Data Centre.

Crystallographic data for **1**: C_15_H_21_NO_4_, *M* = 279.33, *a* = 7.58990(10) Å, *b* = 5.35990(10) Å, *c* = 17.29340(10) Å, *α* = 90°, *β* = 93.0230(10)°, *γ* = 90°, *V* = 702.536(17) Å^3^, *T* = 100(2) K, space group *P*21, *Z* = 2, *μ*(Cu K*α*) = 0.783 mm^-1^, 12970 reflections measured, 2676 independent reflections (*R_int_* = 0.0232). The final *R_1_* values were 0.0263 (*I* > 2*σ* (*I*)). The final *wR*(*F*^2^) values were 0.0666 (*I* > 2*σ* (*I*)). The final *R_1_* values were 0.0264 (all data). The final *wR*(*F*^2^) values were 0.0667 (all data). The goodness of fit on *F*^2^ was 1.112. Flack parameter = 0.01(5).

[1] C. B. Hubschle, G. M. Sheldrick, B. Dittrich, J. Appl. Crystallogr. 44 (2011) 1281–1284.

# **5. Cytotoxicity assay**

Compounds **1**–**6** were evaluated for the cytotoxicity against five human cancer cell lines and normal LO2, including OCVaR, HepG2, HeLa, HT-29, and Hep3B, by a previously reported method [2] The tested cells were purchased from the Cell Bank of Chinese Academy of Science, Shanghai, China. For all cell lines, 5000 cells/ well were seeded into 96-well plates (n = 3) and cultured for 18 h, then exposed to the test compounds at concentrations ranging from 0.064 to 40 *μ*M for 48 h, with *cis*-platin as the positive control. Briefly, 10 μL/well of MTT was added and cultured for another 4 h before removing medium and adding 100 μL of DMSO. The absorbance at 490 nm was detected, and complete medium containing only 0.1% DMSO was defined as 100% and blank well as 0%. The concentration−response relationships were analyzed using Graphpad Prism Software, version 7.0, and IC_50_ values derived using nonlinear regression analysis fit to a logistic equation.

[2] F. Li, S. Lin, S. Zhang, L. Pan, C. Chai, J. C. Su, B. Yang, J. Liu, J. Wang, Z. Hu, Y. Zhang, J. Nat. Prod. 83 (2020) 1931−1938.

# **Figure S1.** ^1^H NMR spectrum of compound **1** (Recorded in methanol-*d*_4_)

# **Figure S2** ^13^C NMR and DEPT spectrum of compound **1** (Recorded in methanol-*d*_4_)

# **Figure S3.** HSQC spectrum of compound **1** (Recorded in methanol-*d*_4_)

# **Figure S4.** HMBC spectrum of compound **1** (Recorded in methanol-*d*_4_)

# **Figure S5** ^1^H–^1^H COSY spectrum of compound **1** (Recorded in methanol-*d*_4_)

# **Figure S6.** NOESY spectrum of compound **1** (Recorded in methanol-*d*_4_)

# **Figure S7.** HRESIMS spectrum of compound **1**


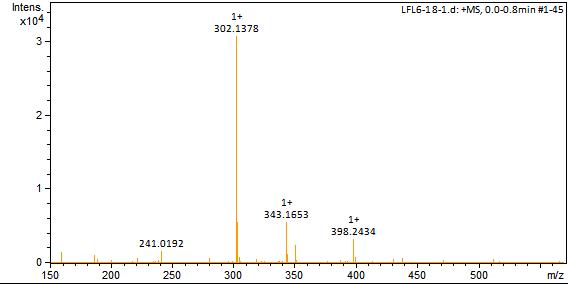


# **Figure S8.** UV spectrum of compound **1**

# **Figure S9**. IR spectrum of compound **1**

# **Figure S10.** ^1^H NMR spectrum of compound **2** (Recorded in methanol-*d*_4_)

# **Figure S11.** ^13^C NMR and DEPT spectrum of compound **2** (Recorded in methanol-*d*_4_)

# **Figure S12.** HSQC spectrum of compound **2** (Recorded in methanol-*d*_4_)

# **Figure S13.** HMBC spectrum of compound **2** (Recorded in methanol-*d*_4_)

# **Figure S14**. ^1^H–^1^H COSY spectrum of compound **2** (Recorded in methanol-*d*_4_)

# **Figure S15.** HRESIMS spectrum of compound **2**

# **Figure S16.** UV spectrum of compound **2**

# **Figure S17**. IR spectrum of compound **2**

# **Figure S18.** ^1^H NMR spectrum of compound **3** (Recorded in methanol-*d*_4_)

# **Figure S19.** ^13^C NMR and DEPT spectrum of compound **3** (Recorded in methanol-*d*_4_)

# **Figure S20.** HSQC spectrum of compound **3** (Recorded in methanol-*d*_4_)

# **Figure S21.** HMBC spectrum of compound **3** (Recorded in methanol-*d*_4_)

# **Figure S22**. ^1^H–^1^H COSY spectrum of compound **3** (Recorded in methanol-*d*_4_)

# **Figure S23.** HRESIMS spectrum of compound **3**

# **Figure S24.** UV spectrum of compound **3**

# **Figure S25**. IR spectrum of compound **3**

1. * Corresponding author. Tel./fax: +86 027 83692311

   E-mail addresses: zhangyh@mails.tjmu.edu.cn (Y. Zhang); hzx616@126.com (Z. Hu); guojieru314@163.com (J. Guo) [↑](#footnote-ref-1)
